# Cost effectiveness analyses of pharmacological treatments in heart failure 

**DOI:** 10.3389/fphar.2022.919974

**Published:** 2022-09-05

**Authors:** Audrey Huili Lim, Nusaibah Abdul Rahim, Jinxin Zhao, S. Y. Amy Cheung, Yu-Wei Lin

**Affiliations:** ^1^ Institute for Clinical Research, National Institutes of Health, Shah Alam, Malaysia; ^2^ Malaya Translational and Clinical Pharmacometrics Group, Department of Clinical Pharmacy and Pharmacy Practice, Faculty of Pharmacy, University of Malaya, Kuala Lumpur, Malaysia; ^3^ Infection and Immunity Program and Department of Microbiology, Biomedicine Discovery Institute, Monash University, Clayton, VIC, Australia; ^4^ Certara (United States), Princeton, NJ, United States

**Keywords:** heart failure, cost effectiveness analysis, pharmacoeconomics, SGLT 2 inhibitor, angiotensin receptor neprilysin inhibitor, ivabradine, vericiguat, omecamtiv

## Abstract

In a rapidly growing and aging population, heart failure (HF) has become recognised as a public health concern that imposes high economic and societal costs worldwide. HF management stems from the use of highly cost-effective angiotensin converting enzyme inhibitors (ACEi) and β-blockers to the use of newer drugs such as sodium-glucose cotransporter-2 inhibitors (SGLT2i), ivabradine, and vericiguat. Modelling studies of pharmacological treatments that report on cost effectiveness in HF is important in order to guide clinical decision making. Multiple cost-effectiveness analysis of dapagliflozin for heart failure with reduced ejection fraction (HFrEF) suggests that it is not only cost-effective and has the potential to improve long-term clinical outcomes, but is also likely to meet conventional cost-effectiveness thresholds in many countries. Similar promising results have also been shown for vericiguat while a cost effectiveness analysis (CEA) of empagliflozin has shown cost effectiveness in HF patients with Type 2 diabetes. Despite the recent FDA approval of dapagliflozin and empagliflozin in HF, it might take time for these SGLT2i to be widely used in real-world practice. A recent economic evaluation of vericiguat found it to be cost effective at a higher cost per QALY threshold than SGLT2i. However, there is a lack of clinical or real-world data regarding whether vericiguat would be prescribed on top of newer treatments or in lieu of them. Sacubitril/valsartan has been commonly compared to enalapril in cost effectiveness analysis and has been found to be similar to that of SGLT2i but was not considered a cost-effective treatment for heart failure with reduced ejection fraction in Thailand and Singapore with the current economic evaluation evidences. In order for more precise analysis on cost effectiveness analysis, it is necessary to take into account the income level of various countries as it is certainly easier to allocate more financial resources for the intervention, with greater effectiveness, in high- and middle-income countries than in low-income countries. This review aims to evaluate evidence and cost effectiveness studies in more recent HF drugs i.e., SGLT2i, ARNi, ivabradine, vericiguat and omecamtiv, and gaps in current literature on pharmacoeconomic studies in HF.

## Introduction

Heart failure (HF) has become recognised as a public health concern that imposes high economic and societal costs worldwide (Di Tanna et al., 2019) as populations age and grow rapidly. HF management stems from the use of highly cost-effective angiotensin converting enzyme inhibitors (ACEi) and β-blockers (BB) to the use of newer drugs such as sodium-glucose cotransporter-2 inhibitors (SGLT2i), angiotensin receptor neprilysin inhibitor (ARNi), ivabradine, vericiguat, and omecamtiv.

Cost of HF management comprises of several components such as hospital management for acute decompensation, physician and outpatient visits, pharmacological management, and home care. However, devised based treatments for mechanical circulatory support, such as implantable cardioverter-defibrillators, as well as new and emerging pharmacological treatment and diagnostics tests have now led to significant increases in HF-related costs. Relatedly, this has placed a huge burden on healthcare systems, and widespread implementation of all potentially beneficial therapies for HF could prove unrealistic for many nations, especially in low- and middle-income countries (LMIC) (Rohde et al., 2013).

In light of recent additions to HF treatment options, it is imperative to understand the economic implications relative to cost effectiveness profiles of the respective pharmacological options. Modelling studies of pharmacological treatments that report on cost effectiveness in HF can help to quantify the relationship between clinical outcomes and help to guide clinical decision making (Rohde et al., 2013).

The objective of cost-effectiveness analysis is to determine if the value of an intervention justifies its cost. More specifically, cost-effectiveness analysis estimates the incremental cost required to improve a selected clinical outcome (e.g., cost per year of life saved, cost per stroke prevented) (Weinstein and Stason, 1977). In estimating the cost-effectiveness ratio, cost is typically measured in dollars. Health benefit, however, may be expressed in a variety of ways. To facilitate comparisons across diseases, health benefit is often quantified as the gain in quality-adjusted life years (QALYs). QALYs are designed to capture the effects of an intervention on both length and quality of life and are calculated by estimating the years of life remaining for a patient following a particular treatment or intervention and weighting each year with a quality-of-life score (on a 0 to 1 scale) (National Institute for Health and Care Excellence, 2022). Specifically, time spent in less-than-ideal health is adjusted downward where the degree of adjustment is determined by the utility for that health state e.g., the utility for an individual’s present health state is 0.5 if the patient equates 2 years of life at their present health state with 1 year of life at ideal health (Rich and Nease, 1999).

In this article, we review evidence and cost effectiveness studies in more recent HF drugs i.e., SGLT2i, ARNi, ivabradine, vericiguat and omecamtiv, and gaps in current literature on pharmacoeconomic studies in HF.

## Types of cost effectiveness analysis

Finite resource must be deployed effectively by policymakers in order for health progression while meeting new challenges and redressing inequities. This requires information on which interventions actually work, their cost, and experience with their implementation and delivery. Cost-effectiveness analysis is a way to examine both the costs and health outcomes of one or more interventions by comparing one intervention to another intervention (or the status quo) and approximating the costs required to gain a unit of a health outcome, e.g., a life year gained or a death prevented. Cost-effectiveness analysis helps identify ways to redirect resources to achieve more by demonstrating not only the utility of allocating resources from ineffective to effective interventions, but also the utility of allocating resources from less to more cost-effective interventions.

### The decision tree

The simplest form of decision analysis models is the decision tree. Each mutually exclusive pathway begins with a “decision node” and goes through “chance nodes” to reach one of several “terminal nodes”. Payoffs are defined at each “terminal node” i.e., costs of healthcare and/or QALY. Incremental cost-effectiveness ratio (ICER) can be estimated by comparing the costs and QALYs for each pathway and treatment option (Thomas and Chalkidou, 2016). Decision trees are most useful when health events are clustered together without repetition, when health events occur quickly or not at all, and when ambiguity of treatment effects are clarified rapidly. A major limitation of a decision tree is its unidirectional flow and as such, may be more suitable for acute disease where all relevant outcomes can be captured in a short time period (Edlin et al., 2015a).

### The Markov model

The Markov model (named after the Russian mathematician Andrei Markov) is a stochastic process that undergoes transitions from one state to another (Li and Zhang, 2009). In the healthcare context, it assumes that patients move between mutually exclusive health states in cycles of a specified length, with death being an absorbing state, because once an individual has entered the state, they must remain there. The probability of a patient remaining in the initial state or moving on into one of the other health states is captured in the model where transitions occur within a defined time period, known as a “Markov cycle”. In each model cycle, individuals have a certain probability of moving between health states, forwards and backwards. The length of model cycle can run for any period of time which allows for modelling up to a full lifetime of a patient (Edlin et al., 2015b; Graves et al., 2016; Komorowski and Raffa, 2016). In the case of heart failure, Markov models would be more ideal than decision trees. The main problem with Markov models is that they become very complicated when more states and more interactions between states are included, especially in the presence of time-dependent probabilities (Carta and Conversano, 2020).

### Micro-simulation

Another decision analysis model is micro-simulation, an individual level state-transition model (Si et al., 2015). Micro-simulation models differ from decision tree or Markov frameworks by using individual level patient history to inform future risk; the other two models use cohort data and associate probability with the “average” patient (Briggs et al., 2006). Unfortunately, micro-simulations were rarely carried out in heart failure cost effectiveness analyses as most health utility estimates were derived from trial data (largely from the same trial for each particular drug). Further advantages and disadvantages of each type of analysis is shown in Table 1.

**TABLE 1:** Types of cost effectiveness analyses and their advantages and disadvantages.

Type of cost effectiveness analysis	Advantages	Disadvantages
Decision tree	• Simple, easy to implement	• Possible overfitting due to over-complex trees that do not generalise the data well
	• Requires little data preparation	• Not ideal for extrapolation as predictions of decision trees are neither smooth nor continuous, but piecewise constant approximations
	• Able to handle both numerical and categorical data	• Decision tree learners create biased trees if some classes dominate
	• Able to handle multi-output problems	
	• Possible to validate a model using statistical tests	
	• Performs well even if its assumptions are somewhat violated by the true model from which the data were generated	
Markov model	• Simplicity and out-of-sample forecasting accuracy	• Inadequate in reflecting decision problems when complexity of decisions increases
	• Generalisability	• Requires data normalisation
	• Based on a formal stochastic process, for which an analytical theory is available	
Micro-simulation	• Simulate the impact of interventions or policies on individual trajectories rather than the deterministic mean response of homogeneous cohorts	• Statistically intensive • Increases likelihood of possible technical errors • Requires data normalisation
	• Individual-level simulation allows the inclusion of stochastic variation in disease progression as well as variation due to individual characteristics	


## New drugs in heart failure and cost-effectiveness review

Cost-effectiveness analyses can help to quantify the relationship between clinical outcomes and the economic implications of new pharmacological treatments in HF. Gathering evidence from these modelling studies will assist in advising clinical decision making in pharmacological treatment, especially due to substantial increase in costs of HF management and widespread implementation of all potentially beneficial therapies for HF could prove unrealistic for many. Table 2 shows a summary of the cost effectiveness studies included in this review.

**TABLE 2:** Summary of cost effectiveness studies included in review.

Drug	Study (first author, year)	Country	Time horizon	Comparator	ICER per QALY	Discount rate	Type of costs	Trial^a^	Type of HF
Dapagliflozin	Gil-Rojas et al. (2021)	Columbia^b^	5 years	SoC	USD$5,946	Cost: 5%; Eff: 5%	Drug acquisition, hospitalisation, emergency visit, adverse events, laboratory procedures	DAPA-HF	HFrEF
	Isaza et al. (2021)	United States	Lifetime	SoC	USD68,300	Cost: 3%; Eff: 3%	Drug acquisition, medications. urgent HF visits, hospitalization, background healthcare costs	DAPA-HF	HFrEF
	Jiang et al. (2021)	China^b^	10 years	SoC	USD$5,541.00	Cost: 5%; Eff: 5%	Drug acquisition, hospitalisation	DAPA-HF	HFrEF
	Krittayaphong and Permsuwan, (2021a)	Thailand^b^	Lifetime	SoC	USD$2,191 for non-diabetics; USD$1,527 for diabetics	Cost: 3%; Eff: 3%	Drug acquisition, medications. Hospitalization, adverse events	DAPA-HF	HFrEF
		Korea			USD$5,277	Cost: 3%	Drug acquisition, medications. Hospitalization		
		Australia			USD$9,980	Eff: 3%			
	Liao et al. (2021a)	Taiwan	15 years	SoC	USD$12,305			DAPA-HF	HFrEF
		Japan			USD$16,705				
		Singapore			USD$23,227				
		United Kingdom			£5,822	Cost: 3.5%; Eff: 3.5%	Drug acquisition, medications, hospitalization, patient review, blood chemistry checking, cardiologist visits, A&E referrals	DAPA-HF	HFrEF
	Mcewan et al. (2020)	Germany	Lifetime	SoC	€ 5,379	Cost: 3%; Eff: 3%		DAPA-HF	HFrEF
		Spain			€ 9,406	Cost: 3%; Eff: 3%		DAPA-HF	HFrEF
	Mendoza, 2021	Philippines^b^	Lifetime	SoC	USD$3,108 - 3,638	Cost: 3%; Eff: 3%	Drug acquisition, hospitalisation, adverse events	DAPA-HF	HFrEF
	Parizo et al. (2021)	United States	Lifetime	SoC	USD$83,650	Cost: 3%; Eff: 3%	Drug acquisition, medications, hospitalization, ambulatory care	DAPA-HF	HFrEF
	Yao et al. (2020)	China^b^	15 years	SoC	USD$3,827.6	Cost: 4.2%; Eff: 4.2%	Drug acquisition, medications, hospitalization	DAPA-HF	HFrEF
	Jiang et al. (2021)	China^b^	10 years	SoC	USD$6,946.69	Cost: 5%; Eff: 5%	Drug acquisition, hospitalisation	EMPEROR-Reduced	HFrEF
		Taiwan			USD$20,508	Cost: 3%	Drug acquisition, medications, hospitalization		
		Japan			USD$24,046	Eff: 3%		
Empagliflozin	Liao et al. (2021b)	South Korea	15 years	SoC	USD$8,846			EMPEROR-Reduced	HFrEF
		Singapore			USD$53,791			
		Thailand^b^			USD$21,543			
		Australia			USD$20,982			
	Reifsnider et al. (2020)	United Kingdom	10 years	SoC	£2,093	Cost: 3.5%; Eff: 3.5%	Drug acquisition, management of acute events, per-episode event costs	EMPA-REG-OUTCOME	HF in T2D
		United Kingdom			€ 20,400	Cost: 3.5%; Eff: 3.5%	Drug acquisition, hospitalisation, adverse events, background medical management, GP visits, outpatient contacts		
	Mcmurray et al. (2018)	Denmark	Lifetime	Enalapril	€ 22,600	Cost: 3%; Eff: 3%		PARADIGM-HF	HFrEF
		Columbia^b^			€ 11,200	Cost: 5%; Eff: 5%			
	Borges et al. (2020)	Portugal	30 years	Enalapril	€ 22,702	Cost: 5%; Eff: 5%	Drug acquisition, HF management, inpatient care, medical visits, adverse events	PARADIGM-HF	HFrEF
	Chin et al. (2020)	Australia	20 years	Enalapril	AUD$40,513	Cost: 5%; Eff: 5%	Drug acquisition, hospitalisation, death	PARADIGM-HF	HFrEF
	Gandjour and Ostwald, (2018)	Germany	Lifetime	Enalapril	€ 23,401	Cost: 3%; Eff: 3%	Drug acquisition, hospitalisation, general healthcare expenditure, laboratory monitoring	PARADIGM-HF	HFrEF
	Gaziano et al. (2020)	United States	Lifetime	Enalapril	USD$21,532	Cost: 3%; Eff: 3%	Drug acquisition, hospitalisation	PARADIGM-HF & PIONEER-HF	HFrEF hospitalisation
					USD$34,727 (*de novo* initiation)	Cost: 1.5%	Drug acquisition, hospitalisation, procedures		
	Grant, 2020	Canada	5 years	Enalapril	USD$40,234 (late initiation)	Eff: 1.5%		PARADIGM-HF	HFrEF
					USD$35,871 (early initiation)				
	King et al. (2016)	United States	Lifetime (40 years)	Enalapril	USD$50959	Cost: 3%; Eff: 3%	Drug acquisition, hospitalisation	PARADIGM-HF	HFrEF
	Krittayaphong and Permsuwan, (2018)	Thailand^b^	Lifetime	Enalapril	USD$4,857.11	Cost: 3%; Eff: 3%	Drug acquisition, hospitalisation	PARADIGM-HF	HFrEF
	Krittayaphong and Permsuwan (2021b)	Thailand^b^	Lifetime	Enalapril	USD$3,451.26	Cost: 3%; Eff: 3%	Drug acquisition, hospitalisation	PARADIGM-HF & PIONEER-HF	Acute decompensated HF
Sacubitril/Valsartan	Liang et al. (2018)	Singapore	10 years	Enalapril	USD$55,198	Cost: 3%; Eff: 3%	Drug acquisition, hospitalisation, readmissions	PARADIGM-HF	HFrEF
	Park, 2019	South Korea	Lifetime	Enalapril	USD$11,970	Cost: 5%; Eff: 5%	Drug acquisition, hospitalisation, monitoring, adverse events, terminal care	PARADIGM-HF	HFrEF
	Perera et al. (2019)	Australia	Lifetime	Enalapril	AUD$77,889	Cost: 5%; Eff: 5%	Drug acquisition, hospitalisation, death	PIORNEER-HF	Acute decompensated HF
	Ramos, 2017	Netherlands	Lifetime	Enalapril	€ 17,600	Cost: 4%; Eff: 1.5%	Drug acquisition, HF management, hospitalisation, adverse events, informal care, traveling expenses	PARADIGM-HF	HFrEF
	Sandhu et al. (2016)	United States	Lifetime	Lisinopril	USD$44531 (NYHA Class II); USD$58194 (NYHA Class III)	Cost: 3%; Eff: 3%	Drug acquisition, hospitalisation, adverse events	PARADIGM-HF	HFrEF
	Van Der Pol et al. (2017)	Netherlands	30 years	Enalapril	€ 19,133	Cost: 4%; Eff: 1.5%	Drug acquisition, hospitalisation, elderly care and GP costs	PARADIGM-HF	HFrEF
	Wu et al. (2020)	China	10 years	Enalapril	USD$2,480.67	Cost: 3.5%; Eff: 3.5%	Drug acquisition, hospitalisation, outpatient visit, coay ratio for inpatient, cost of events, readmssion	PARADIGM-HF	HFrEF
	Zakiyah et al. (2021)	Indonesia^b^	10 years	Enalapril	USD$1,890	Cost: 3%; Eff: 3%	Drug acquisition, hospitalisation	PARADIGM-HF	HFrEF
	Zanfina, 2017	Switzerland	Lifetime	Enalapril	CHf25684	Cost: 3%; Eff: 3%	Drug acquisition, hospitalisation, management of HF by physicians, background drug therapy, adverse events, titration	PARADIGM-HF	HFrEF
	Zueger et al. (2018)	United States	5 years	Enalapril	USD$14,3891	Cost: 3%; Eff: 3%	Drug acquisition, hospitalisation	PARADIGM-HF	HFrEF
Ivabradine	Adena, 2018	Australia	10 years	SoC	AUD$14,905	Cost: 5%; Eff: 5%	Drug acquisition, medications. Hospitalization	SHIFT	Chronic HF
	Griffiths, 2014	United Kingdom	Lifetime	SoC	£8,498 for HR ≥75 bpm	Cost: 3.5%	Drug acquisition, hospitalization	SHIFT	Chronic HF
					£13,764 for HR ≥ 70bpm	Eff: 3.55%			
	Kansal, 2016	United States	10 years	SoC	USD$24,920	—	Drug acquisition, specialist visits, hospitalization, adverse events	SHIFT	Chronic HF
	Kourlaba, 2014	Greece	Lifetime	SoC	€ 9,986	Cost: 3.5%; Eff: 3.5%	Drug acquisition, hospitalisation, HF management	SHIFT	Chronic HF
	Krittayaphong et al. (2019)	Thailand^b^	Lifetime	SoC	USD$6,515	Cost: 3%; Eff: 3%	Drug acquisition, medications. hospitalization	SHIFT	HFrEF
	Taheri, 2018	Iran	10 years	SoC	USD$5,437	Cost: 7.2%; Eff: 5%	Drug acquisition, hospitalisation, medical care, HF management, adverse events	SHIFT	Chronic HF
Vericiguat	Alsumali, 2021	United States	30 years	SoC	USD$82,448	Cost: 3%; Eff: 3%	Drug acquisition, heart failure hospitalization, routine care, and terminal care	VICTORIA	HFrEF

a Name of trials included in this list in included in Supplementary Table S1.

b Low- or middle-income country.

Abbreviations: ICER, Incremental Cost Effectiveness Ratio; QALY, Quality Adjusted Life Years: SoC, Standard of Care; HFrEF, Heart Failure with Reduced Ejection Fraction; HF, Heart Failure; T2D, Type 2 Diabetes; Eff, Effect.

### Sodium-glucose cotransporter-2 inhibitors

SGLT2i have recently risen in popularity in their use in HF. Several trials have been carried out to address this important knowledge gap, namely DAPA-HF, PRESERVED-HF, EMPA-REG OUTCOME, EMPEROR-Preserved, and SOLOIST-WHF. Multiple systematic reviews and meta-analysis have shown that SGLT2i reduce all-cause and cardiovascular mortality in HFrEF across subgroups of sex, age, and race, regardless of baseline diabetes status (Zannad et al., 2020; Cardoso et al., 2021; Tsampasian et al., 2021).

Dapagliflozin was the first SGLT2i approved for the treatment of HFrEF. Results from DAPA-HF have been used in multiple cost effectiveness studies (Mcewan et al., 2020; Yao et al., 2020; Krittayaphong and Permsuwan, 2021a; Liao et al., 2021b; Gil-Rojas et al., 2021; Isaza et al., 2021; Jiang et al., 2021; Parizo et al., 2021), of which two were multinational health economic analysis. One was simulated in Germany, Spain and United Kingdom (Mcewan et al., 2020), the other in the Asia-Pacific region (Korea, Australia, Taiwan, Japan, and Singapore) (Liao et al., 2021b). The Kansas City Cardiomyopathy Questionnaire (KCCQ) total symptom score was used for quality of life measure in DAPA-HF. McEwan et al. reported treatment with dapagliflozin increased life-years and QALYs by 0.58 and 0.48 respectively, and reduced lifetime hospitalisations for HF by 105 events per 1,000 patients (Mcewan et al., 2020). The threshold for willingness-to-pay used was £20,000/QALY where more than 90% of simulations were cost-effective. Isaza et al. reported an ICER of $68,300/QALY in the United States of America (USA) (Isaza et al., 2021) but Krittayaphong and Permsuwan reported an ICER of $2,191/QALY in non-diabetics and $1,527/QALY in diabetics. This substantial difference highlights the importance of local settings when calculating cost effectiveness. ICERs based on United States settings have a tendency to be higher due to higher drug unit costs (Hewitt et al., 2018). A study from China showed that dapagliflozin had a lower ICER than empagliflozin when compared to standard treatment in HFrEF (Jiang et al., 2021), indicating dapagliflozin may be the preferred choice of SGLT2i in HFrEF.

Fewer cost effectiveness studies have been conducted on other SGLT2i (Reifsnider et al., 2020; Liao et al., 2021a). Reifsnider et al. showed that empagliflozin had an ICER of £2,093/QALY using data from HF subpopulation data from the EMPA-REG OUTCOME trial (Reifsnider et al., 2020). Liao et al. used transitional probabilities derived from the EMPEROR-Reduced trial to demonstrate ICER of $20,508, $24,046, $8,846, $53,791, $21,543, and $20,982 in Taiwan, Japan, South Korea, Singapore, Thailand, and Australia respectively (Liao et al., 2021a).

Despite mounting evidence of the use of SGLT2i in HFrEF, there has been a lack of evidence of its use in heart failure with preserved ejection fraction (HFpEF) which accounts for the majority of all HF in the community. The EMPEROR-Preserved trial was designed to address this knowledge gap, followed by the PRESERVED-HF, SOLOIST-WHF, SCORED, and DELIVER trials. With the exception of DELIVER (which is expected to be published in 2022), the other trials have delivered promising results of the use of SGLT2i in HFpEF (Bhatt et al., 2020a; Bhatt et al., 2020b; Anker et al., 2021; Nassif et al., 2021; Packer et al., 2021; Solomon et al., 2021). DELIVER was designed to complement DAPA-HF which assessed the efficacy of dapagliflozin in patients with HFrEF, specifically in patients with and without diabetes. The results of both studies will be pooled to assess the effects of dapagliflozin across the spectrum of ejection fraction to allow for a wide range of patients with mildly reduced ejection fraction (Solomon et al., 2021).

Congestion and impaired renal function are hallmarks of all types of heart failure, including HFpEF, and appear to be ameliorated by SGLT2i. Therefore, SGLT2i may have beneficial effects across the range of LVEF by improving kidney function as chronic kidney disease is a major risk factor for adverse outcomes in HFpEF. SGLT2i also appear to improve diastolic function, reduce obesity, and visceral fat (including epicardial fat), reduce arterial stiffness, improve endothelial function, and reduce inflammation, all of which are important mechanisms of HFpEF pathogenesis (Solomon et al., 2021).

In line with recent NICE guidance (National Institute for Health and Care Excellence, 2021), the use of SGLT2i in the HFrEF population is beginning to increase. Hooper et al. (2021) found 85% of non-diabetic eligible patients were not treated with SGLT2i but predicted this figure is likely to fall significantly over the next year as awareness of this new treatment increases and local guidelines include this class of agent. Although the FDA has recently approved the use of empagliflozin in HFpEF, there is a lack of guideline-directed therapy for patients with HF with LVEF >40%.

### Sacubitril/valsartan

Sacubitril/valsartan is the first angiotensin receptor neprilysin inhibitor (ARNi) for the treatment of HFrEF. PARADIGM-HF was a pivotal clinical trial that compared the effects of sacubitril/valsartan with enalapril and showed clinically relevant and statistically significant reduction in CV mortality and morbidity in patients with HFrEF (Krittayaphong and Permsuwan, 2018; Liu et al., 2021). This was followed by several smaller trials such as TITRATION, PRIME HF, EVALUATE-HF, PROVE-HF, PIONEER-HF, and TRANSITION. These trials highlight the range of use for sacubitril/valsartan, not only in chronic HF but also in the acute HF setting, suggesting the continuum of use across the outpatient and inpatient settings. However, CEAs have only been conducted in chronic HFrEF and acute decompensated HF.

PARADIGM-HF was a large, multicentre trial in the ambulatory setting while PIONEER-HF was designed specifically designed to assess outcomes in the acute in-hospital setting. This led to differing utility values from both trials and hence differing ICERs despite accounting for similar costs by Chin et al. (2020) and Perera et al. (2019). In this case, the studies by Perera et al. (2019), Gaziano et al. (2020), and Krittayaphong and Permsuwan (2021b) were the only ones which investigated acute decompensated HF, of which only the study from Thailand showed ICER below their local threshold.

A real-world effectiveness evaluation of sacubitril/valsartan by Proudfoot et al. (2021) indicated that most studies reported superior efficacy of sacubitril/valsartan in reducing the risk of HF hospitalisations, all-cause hospitalisations, and all-cause mortality as compared to standard of care. A significant improvement in NYHA functional class was observed, with studies reporting improvement in health-related quality of life (HRQoL). Although current guidelines for HF recommend ACEi/ARB as first line treatment, a systematic review by Tromp et al., 2022) has recently found that the combination of ARNi showed a smaller probability of all-cause mortality compared to ACEi/BB.

Despite regulatory approval in 2015, there has been poor uptake of sacubitril/valsartan for clinical use. As the drug acquisition cost of sacubitril/valsartan is higher than that of an ACEi, an estimation of expected costs and benefits is necessary for reimbursement by national payers in order to determine value for money. Various cost effectiveness analyses for sacubitril/valsartan in HF showed that the ICERs ranged from $1,890/QALY (Zakiyah et al., 2021) to $14,3891/QALY (Zueger et al., 2018). Although ICERs from most studies were below the implemented country-specific thresholds with the exception of Thailand and Singapore (King et al., 2016; Sandhu et al., 2016; Ademi et al., 2017; Van Der Pol et al., 2017; Gandjour and Ostwald, 2018; Krittayaphong and Permsuwan, 2018; Liang et al., 2018; Mcmurray et al., 2018; Zueger et al., 2018; Borges et al., 2020; Gaziano et al., 2020; Zakiyah et al., 2021), they were still less cost effective than dapagliflozin and empagliflozin. These studies used standard drug treatment of enalapril/lisinopril as comparators. With limited healthcare resources, compared with enalapril, sacubitril/valsartan may not be considered as a cost-effective strategy for chronic HF in Singaporean and Thai healthcare perspectives (Liu et al., 2021).

### Ivabradine

Ivabradine is a selective I_f_ channel blocker that inhibits the pacemaker current of the sinoatrial node cells, which results in a reduced heart rate without affecting or lowering of blood pressure, or modification of cardiac contractility, or adverse modulating on the sympathetic system (Das et al., 2017; Badu-Boateng et al., 2018). The results from the SHIFT trial indicated that ivabradine therapy reduced CV death or hospitalisation, increased life expectancy and improved life quality in HFrEF. A range of economic evaluation studies of ivabradine simulated ICERs ranging from $10,616/QALY in Thailand (Krittayaphong et al., 2019) to $55,600/QALY in United States (Rashki Kemmak et al., 2021), indicating that ivabradine is more cost effective than sacubitril/valsartan but less than empagliflozin and dapagliflozin. In this case, SGLT2i should be added on to HFrEF treatment before ivabradine.

### Vericiguat

Vericiguat is a novel oral soluble guanylate cyclase stimulator which enhances the cyclic guanosine monophosphate (cGMP) pathway by directly stimulating soluble guanylate cyclase through a binding site independent of nitric oxide (Armstrong et al., 2020b; Lombardi et al., 2021). In the VICTORIA trial, patients with HFrEF were found to have lower CV death and hospitalisation. Cost effectiveness models based on data from this trial compared vericiguat to standard of care, leading to an ICER of $82,448/QALY. This placed vericiguat generally within the same cost effectiveness region as sacubitril/valsartan.

In patients with HFpEF, there have been contradicting evidence from two different trials, where vericiguat improved the pre-specified exploratory endpoint of KCCQ Clinical Summary Score by mean 19.3 points in the SOCRATES-PRESERVED (Pieske et al., 2017) but the VITALITY-HFpEF found that vericiguat did not improve the physical limitation score of the KCCQ (Armstrong et al., 2020a). Although some differences in characteristics of the study population may have led to this difference in findings and the lack of benefit with nitrates and phosphodiesterase inhibitors suggest that direct soluble guanylate cyclase stimulation with vericiguat is ineffective, further study in this area is warranted before excluding its use in HFpEF.

### Omecamtiv

Omecamtiv mecarbil is a direct cardiac myosin activator currently being studied in the GALACTIC-HF trial. It increases systolic ejection time and stroke volume, improves ventricular remodelling, and decreases natriuretic peptide concentrations in patients with HFrEF. Post hoc analysis of results from the GALACTIC-HF trial showed that omecamtiv mecarbil may provide a clinically meaningful reduction in time to first HF event or CV death in patients with severe HF (Felker et al., 2022). Currently, there are plans for FDA approval of the drug in the coming year (Tilyou, 2021). Cost effectiveness analyses based on results from the GALACTIC-HF trial will be useful in order to quantify the benefit of omecamtiv mecarbil once it has received regulatory approval.

## Gaps in studies and potential for future development

**FIGURE 1:**
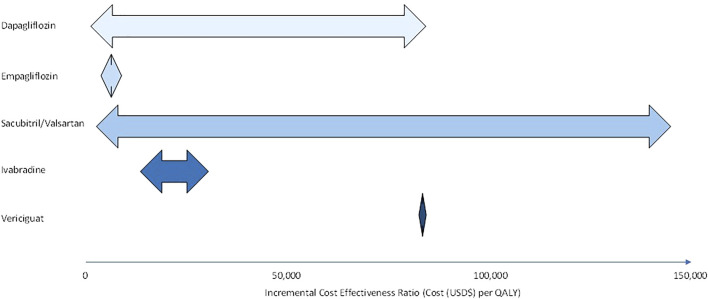
Range of incremental cost effectiveness ratios for dapagliflozin, empagliflozin, sacubitril/valsartan, ivabradine, and vericiguat in heart failure (HFrEF and HFpEF) patients.

Of all the pharmacological treatment measures reviewed in this article, SGLT2i have the most extensive cost effectiveness analyses. Evaluation of the aforementioned cost effectiveness analyses shows that sacubitril/valsartan has the greatest range of ICERs (Figure 1). Baseline CV mortality risk score is the most commonly evaluated model drive in pharmacoeconomic evaluation of HF. It should be noted that there are few studies that evaluate treatment time horizon and hospitalisation costs. Furthermore, there is clearly a lack of studies that model rehospitalisation changes explicitly, only one study in this review included hospital readmissions in its cost evaluation (Wu et al., 2020). This is empirical in the case of HF as patients with HF who have previously been hospitalised have elevated rehospitalisation rates and increased care costs (Rohde et al., 2013).

Evaluation of the economic and societal implications of HF should take into account indicators of (re) hospitalisation which can provide crucial information beyond classification instruments and offer further details about patient profiles. However, one should be cautious with the use of generalised indicators for hospitalisation in a model structure due to potential for bias, as skewing in observations and related costs could occur in cases of multiple hospital visits (Di Tanna et al., 2019). The use of urgent heart failure visits as an endpoint could also be beneficial for modelling purposes as these visits which require intravenous diuretic therapy have been a component of the primary endpoint of several prior heart failure trials, including DAPA-HF, and have proven to be both prognostically similar to heart failure hospitalisations and similarly discriminative of treatment effects in several trials (Solomon et al., 2021).

Social perspectives as well as other costs can affect the cost effectiveness of various pharmacological treatment, especially if the drug of choice is costly, and these costs vary between countries. In evaluating cost effectiveness analyses, the threshold chosen by each country can have a significant impact on these results. Country income levels are likely to influence the ratio between the consumption value of health and threshold for health due to varying healthcare budgets. Limitations in increase of tax revenues are often a reason for constrained healthcare budgets (Woods et al., 2016), especially for LMICs. As drug costs differ in each country, the relative ratio of the new drug against the comparator tend to fluctuate. However, the disparity is more apparent in LMICs where low-cost generics of standard therapy (e.g., ACEi) are substantially cheaper that these newer drugs, and as such it may not be ideal to compare cost effectiveness analyses from high income countries to that of LMICs.

The disparity in choice of time horizons used in cost effectiveness studies reflects some variability in model structure. When simulated horizons are prolonged, respondent ICER tend to decrease (Yao et al., 2020). Variation in treatment time horizons affects the ICER as one that is too short may be unable to capture the benefit of the medication. For example, Zueger et al. (2018) showed an ICER of USD$143891 for sacubitril/valsartan when compared with enalapril over 5 years while King et al. (2016) showed an ICER of USD50959 over a lifetime (approximated over 40 years). Similar costs were taken into account for both studies, the main difference was he length of the time horizon. This should also be taken into account when evaluating cost effectiveness analyses. Moreover, there has been a shift in trend away from cost-effectiveness analysis carried out using clinical trial data (or extrapolations from these) towards a modelling-based approach for example using Markov modelling. The use of a Markov model in this case is more ideal as heart failure has a continuous risk over time and has the possibility of more than one major event (e.g., (re) hospitalization, death). The use of deterministic sensitivity/scenario analysis and/or probabilistic sensitivity analysis is also essential to assess in detail the parameter uncertainty and the impact of key variables in the cost-effectiveness profiles.

One of the limitations of this review is we are unable to address the cost effectiveness of ivabradine, vericiguat, and omecamtiv appropriately due to the lack of studies on these newer drugs. As such, there is a need to address this gap in knowledge as well as looking into CEAs of sacubitril/valsartan in other conditions of HF aside from chronic HF and acute decompensated HF.

Furthermore, cost effectiveness studies that evaluate pharmacological therapy in HFpEF remains unexplored. As HF patients with less severe conditions and greater ejection fraction may obtain less benefit from add-on therapy, the cost-benefit ratio of using expensive pharmacological therapy may be smaller, hence greater ICER. As such, some drugs may only be cost effective in certain subgroups of patients.

HF treatment may also be guided by testing for B-type natriuretic peptide (BNP). BNP is a cardiac neurohormone secreted from the ventricles in response to ventricular volume expansion and pressure overload (Moe, 2006), whereby its increased presence in the blood is indicative of a higher risk of heart attack, heart failure or death (Lainchbury et al., 2009; Pfisterer et al., 2009; Porapakkham et al., 2010). Many clinical studies now recommend the use of BNP testing for diagnosing acute HF instead of the common and non-invasive method of echocardiography (Doust et al., 2006; Yoo, 2014). However, there is uncertainty about the cost effectiveness of BNP testing. A systematic review by Jafari et al. (2018) concluded that the use of BNP testing in patients with heart failure may reduce cost compared to the symptom-based clinical care and increase QALY. Treatment of HF should not only take into account cost of treatment but also possible testing for markers such as BNP which may improve cost effectiveness of treatment. However, it is to be noted that there has been a lack of cost effectiveness studies of BNP testing in LMICs, hence, an area to be further investigated.

## Conclusion

In order for more precise analysis on cost effectiveness analyses, it is necessary to take into account the income level in various countries as it is certainly easier to allocate more financial resources for the intervention, with greater effectiveness, in high- and middle-income countries than in low-income countries. Although cost effectiveness analysis on newer pharmacological treatments such as SGLT2i, ARNi, ivabradine, vericiguat, and omecamtiv in HFrEF have been established, there is still a paucity of evidence for their use in HFpEF.
