# Supplementary material for: Cost effectiveness analyses of pharmacological treatments in heart failure
Source: Front Pharmacol. 2022 Sep 5;13:919974. doi: 10.3389/fphar.2022.919974 (PMC9483981; doi:10.3389/fphar.2022.919974)
Supplement: Supplementary file 1 [file Table1.docx]

Supplementary Table 1: List of trials included

| Name | Title |
| --- | --- |
| DAPA-HF | Dapagliflozin and Prevention of Adverse Outcomes in Heart Failure |
| DELIVER | Dapagliflozin Evaluation to Improve the Lives of Patients with Preserved Ejection Fraction Heart Failure |
| EMPA-REG OUTCOME | Empagliflozin Cardiovascular Outcome Event Trial in Type 2 Diabetes Mellitus Patients |
| EMPA-TROPISM | Are the "Cardiac Benefits" of Empagliflozin Independent of Its Hypoglycemic Activity? |
| EMPEROR-Preserved | Empagliflozin Outcome Trial in Patients with Chronic Heart Failure with Preserved Ejection Fraction |
| EVALUATE-HF | Study of Effects of Sacubitril/Valsartan vs. Enalapril on Aortic Stiffness in Patients With Mild to Moderate HF With Reduced Ejection Fraction |
| GALACTIC-HF | Registrational Study With Omecamtiv Mecarbil (AMG 423) to Treat Chronic Heart Failure With Reduced Ejection Fraction |
| PARADIGM-HF | Efficacy and Safety of LCZ696 Compared to Enalapril on Morbidity and Mortality of Patients With Chronic Heart Failure |
| PIONEER-HF | Comparison of Sacubitril/Valsartan Versus Enalapril on Effect on NT-proBNP in Patients Stabilized From an Acute Heart Failure Episode. |
| PRESERVED-HF | Dapagliflozin in Preserved Ejection Fraction Heart Failure |
| PRIME-HF | Predischarge Initiation of Ivabradine in the Management of Heart Failure |
| PROVE-HF | Effects of Sacubitril/Valsartan Therapy on Biomarkers, Myocardial Remodeling and Outcomes. |
| SCORED | Effect of Sotagliflozin on Cardiovascular and Renal Events in Patients With Type 2 Diabetes and Moderate Renal Impairment Who Are at Cardiovascular Risk |
| SHIFT | Effects of Ivabradine on Cardiovascular Events in Patients With Moderate to Severe Chronic Heart Failure and Left Ventricular Systolic Dysfunction. A Three-year International Multicentre Study |
| SOCRATES-PRESERVED | Safety and Efficacy Study of Four Dose Regimens of BAY1021189 in Patients With Heart Failure and Preserved Ejection Fraction Suffering From Worsening Chronic Heart Failure |
| SOLOIST-WHF | Effect of Sotagliflozin on Cardiovascular Events in Patients With Type 2 Diabetes Post Worsening Heart Failure |
| TITRATION | Safety and Tolerability of Initiating LCZ696 in Heart Failure Patients |
| TRANSITION | Comparison of Pre- and Post-discharge Initiation of LCZ696 Therapy in HFrEF Patients After an Acute Decompensation Event |
| VICTORIA | A Study of Vericiguat in Participants With Heart Failure With Reduced Ejection Fraction |
| VITALITY- HFpEF | Patient-reported Outcomes in Vericiguat-treated Patients With HFpEF |
